# Electroacupuncture Attenuates Ovalbumin-Induced Allergic Asthma via Modulating CD4^+^CD25^+^ Regulatory T Cells

**DOI:** 10.1155/2012/647308

**Published:** 2012-05-10

**Authors:** Youngjoo Kwon, Sung-Hwa Sohn, Gihyun Lee, Youngeun Kim, Hyejung Lee, Minkyu Shin, Hyunsu Bae

**Affiliations:** ^1^Department of Physiology, College of Oriental Medicine, Kyung Hee University, Seoul 130-701, Republic of Korea; ^2^Acupuncture and Meridian Science Research Center, Kyung Hee University, Seoul 130-701, Republic of Korea

## Abstract

A mouse pulmonary hypersensitivity experimental model that mimics human asthma was developed, and electroacupuncture (EA) treatment was shown to reduce allergic inflammatory processes. In addition, we also assessed whether the beneficial effects of EA on allergic asthma could be correlated with CD4^+^CD25^+^Foxp3^+^ regulatory T cells (Treg). Cellular profiles and histopathologic analysis demonstrated that peribronchial and perivascular inflammatory cell infiltrates were significantly decreased in the EA-treated groups when compared to the OVA and anti-CD25 Ab-injected (Treg depletion) groups. Furthermore, total BAL cells were reduced in the EA groups when compared to other groups. Interestingly, the population of CD4^+^CD25^+^Foxp3^+^Tregs in pneumonocytes increased in EA-treated group when compared to OVA and Treg depletion groups. These results imply that EA stimulation at ST 36 may affect CD4^+^CD25^+^Foxp3^+^ Treg in an OVA-induced experimental model and may enhance Treg function by suppressing other T cells and limiting the immune response.

## 1. Introduction

Acupuncture is the clinical insertion and manipulation of thin needles into specific body sites, the so-called acupoints on the meridian, and is based on the ancient theory of oriental medicine. This process is believed to elicit profound psychophysical responses by harmonizing or balancing the energy and blood flow through the body. EA is a modified technique of acupuncture that requires electrical stimulation. Several studies have reported that EA stimulation is effective for the treatment of allergic disorders caused by an imbalance of the Th1/Th2 cell response, such as asthma [1–4]. Allergic asthma is an inflammatory process driven by inappropriate Th2 immune responses against otherwise innocuous environmental allergens [5–7], and also involves complex neuroimmune deregulation that promotes bronchial infiltration of inflammatory leukocytes, which results in exacerbated mucus production, epithelial damage, airway hyperresponsiveness (AHR) and tissue remodeling. This response is characterized by airway infiltration of diverse effectors cells such as monocytes, mast cells, neutrophils, T lymphocytes, and eosinophils [8, 9]. The underlying process that drives and maintains the asthmatic inflammatory process appears to be an imbalance of the equilibrium between the Th1 and Th2 immune response types, with a predominance of Th2 [10]. Our previous study showed that EA reduces IgE in BALB/c mice immunized with 2,4-dinitrophenylated keyhole limpet protein (DNP-KLH) through the suppression of Th2 cytokines [11, 12]. And these effects were mediated by *α*-adrenoreceptor [12]. Recently, regulatory T (Treg) cells are shown to be key downregulatory cells that are capable of preventing allergic sensitization and progression of allergic disease, including asthma. Treg cells are important components of the homeostasis of the immune system, since impaired CD4^+^CD25^+^ T-cells activity can cause autoimmune diseases and allergy [7, 13]. Especially, Foxp3 is a transcription factor that is predominantly expressed in CD4^+^CD25^+^ Treg cells and is a master regulator of the development and function of Treg cells [14–16]. Tregs have been found to lose Foxp3 expression when converted into proinflammatory helper cells or CD4^+^ follicular helper cells [17–19]. In addition, Treg cells actively control or suppress the function of other cells, generally in an inhibitory fashion, and play an essential role in the maintenance of peripheral self-tolerance by preventing the activation and proliferation of autoreactive T cells. These cells appear to control the development of autoimmune diseases and transplant rejection and may also play a critical role in controlling allergic diseases including atopic asthma [20–22]. Given the reasonable capacity of Treg cells to suppress the diseases-promoting immune responses, enhancing Tregs function may be an attractive therapeutic strategy for the treatment of allergic diseases and asthma. These previous observations, together with other reports concerning the anti-inflammatory action of acupuncture, led us to examine the effect of acupuncture on an experimental asthma model to better understand some of the immunologic effects of acupuncture. In this study, we also evaluated the antiallergic asthma mechanisms of EA on OVA-induced allergic asthma mice.

## 2. Materials and Methods

### 2.1. Animals

Balb/c female littermates (6 to 8 weeks of age, weighing 20–25 g) were purchased from Orient Bio (Seoungnam, South Korea). Foxp3^+^EGFP Balb/c (C. Cg-Foxp3^tm2Tch^/J) mice were purchased from The Jackson Laboratory (Bar Harbor, ME, USA). All mice were kept under pathogen-free conditions using air conditioners and a 12-h light/dark cycle. In addition, all mice had free access to food and water during the experiments. The study was conducted according to the Rules for Animal Care and the Guiding Principles for Animal Experiments Using animal by the University of Kyung Hee Animal Care and Use Committee and in accordance with the recommendations of the Weatherall report, “The use of non-human primates in research.” The protocols for the experimental procedures were approved by the Animal Welfare and Animal Care Committee of the University of Kyung Hee Animal Care and Use Committee (KHUASP(SE)-09-017).

### 2.2. Induction of Allergic Asthma

Female mice 6–8 weeks of age were sensitized by intraperitoneal (i.p) injection on days 0 and 14 with 100 *μ*g of ovalbumin (OVA) (Sigma-Aldrich, St. Louis, MO, USA) precipitated with 20 mg of aluminum hydroxide in 100 *μ*L of PBS. The mice were then challenged by administering 1% OVA in 50 *μ*L PBS or PBS directly into the nostrils using a micropipetor on days 21–30. Negative control mice were sensitized and challenged with PBS alone. On day 31, the mice were sacrificed, and various tissues were collected for analyses.

### 2.3. Experimental Protocol and Design

**Figure 1:**
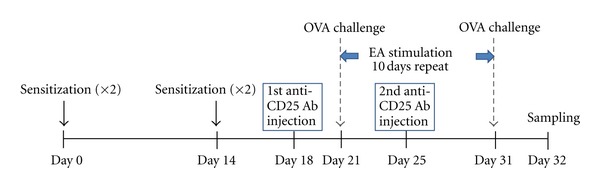
Schematic diagram of the experimental protocol. On days 0 to 14, mice were divided into control (PBS-treated) and OVA-induced asthmatic groups. On day 18, OVA-induced asthmatic group was separated into 4 groups (OVA-immunized group; EA-treated after OVA induction group; anti-CD25 antibody injection after OVA induction group; EA-treated after OVA induction; anti-CD25 antibody injection group). Mice were immunized i.p. with 0.25 mg of anti-CD25 antibody 3 days before intranasal challenge. On day 21, all of OVA-induced asthmatic groups received 1% OVA in 50 *μ*L PBS or PBS intranasally and after 10 minutes, EA treatments at ST36 were applied for another 10 minutes. Acupuncture stimulations were repeated for ten days consecutively after intranasal challenge.

On days 0 to 14, mice were divided into control (PBS-treated) and OVA-induced asthmatic groups. On day 18, OVA-induced asthmatic group was separated into 4 groups (OVA-treated group; OVA + EA group; OVA and anti-CD25 antibody injection group; OVA and anti-CD25 antibody injection + EA group). Mice were treated i.p. with 0.25 mg of anti-CD25 antibodies 3 days before intranasal challenge. On day 21, all OVA-induced asthmatic groups received 1% OVA in 50 *μ*L PBS or PBS intranasally, and after 10 minutes, EA stimulation at ST36 was performed. EA stimulation was applied for 10 minutes and repeated for ten consecutive days for 10 min. after intranasal challenge (Figure 1).

### 2.4. Depletion of CD4^+^CD25^+^ T Cells *In Vivo*

**Figure 2:**
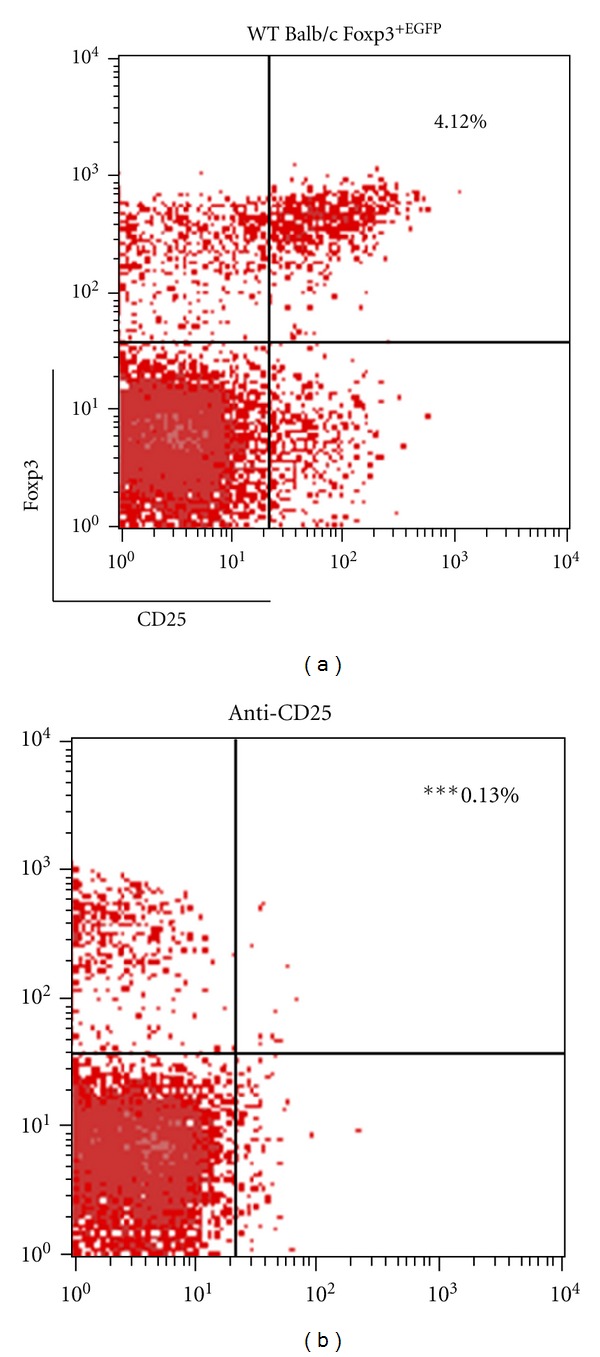
Confirmation of CD4^+^CD25^+^ Treg cells depletion. PBS treatment alone; anti-CD25 antibody injection into WT Balb/c Foxp3^+ EGFP^ (anti-CD25). Balb/c mice received injections of 0.25 mg of anti-CD25 antibody after OVA sensitization. The efficacy of CD4^+^CD25^+^ Treg cells depletion was confirmed by flow cytometry analysis using, PE-anti-mouse CD25 and Foxp3^+ EGFP^. Data are shown as mean ± S.E.M. (***P < 0.001 versus control *n* = 4).

Anti-mouse CD25 rat IgG1 (anti-CD25; clone PC61) were generated in-house from hybridomas obtained from American Type Culture Collection (Manassas, VA, USA). A dose of 0.25 mg of the anti-CD25 antibody was injected 3 days before ovalbumin i.p. administration. The efficacy of CD4^+^CD25^+^ Treg cell depletion was confirmed by flow cytometry analysis, using PE-anti-mouse CD25 and fluorescein isothiocyanate-anti-mouse CD4 (Figure 2). 

### 2.5. EA Stimulation at ST 36

 Acupoint ST36 has been used to treat asthma. Acupoint ST36 is located 5 mm below and lateral to the anterior tubercle of the tibia. Electrical stimulation was applied to ST36 using two outlets via two needles. The needles (length 3.0 cm; diameter 0.20 mm) were inserted perpendicular to the skin, 5 mm from each other, and about 5 mm to one side of the anterior tibial muscle. An electrical pulse of 3–5 V for a, duration 0.25 ms and frequency of 1 Hz was delivered from an EA stimulator, PG-306 (YoungMok, Japan). The intensity of stimulation was determined to be the minimum voltage that caused moderate muscle contraction. EA stimulation was applied for 10 minutes. Control mice were restrained in acrylic holders without EA stimulation for 10 min to normalize stresses across the groups. Acupuncture stimulations were repeated for ten consecutive days for 10 min after intranasal challenge.

### 2.6. Fluorescence-Activated Cell Sorting and Flow Cytometric Analysis

Lungs were removed, washed with PBS to remove blood, minced and erythrocyte were lysed in 1% RBC lysis buffer (BD, Pharmingen) for 5 minutes. After RBC lysis, the lung tissues were forced through a 25 *μ*m cell strainer. Cells were washed three times and resuspended in FCM buffer (PBS with 2% FBS and 0.1% NaN_3_). Single-cell suspensions and pneumonocytes obtained from Foxp3EGFP Balc/C mice were labeled with anti-CD4-APC and anti-CD25-PE mAb using standard staining methods, and the percentage of cells stained with a particular reagent was analyzed by FACSCalibur using the CellQuest software (BD Bioscience).

### 2.7. Bronchoalveolar Lavage (BAL)

BAL was collected by infusion and extraction of 1 mL of ice-cold PBS. This was repeated three times, and the lavages pooled (mean volume, ±  2.0 mL). Recovered BAL (70–80%) was centrifuged at 13000 rpm for 10 min. The cell pallets were resuspended in 1 mL PBS, and the cells were adhered to glass slides using cytocentrifugation. Total viable cell counts were determined in a hemocytometer using trypan blue exclusion. Differential counts of eosinophils, neutrophils, lymphocytes, and macrophages were determined on cytospin smears of BALF samples (5 × 10^5^/200 *μ*L cells) from individual mice stained with Diff-Quick staining (Life Technologies, Auckland, New Zealand) after counting 500 cells. The BAL fluid was then centrifuged and the supernatants were kept at −70°C. Results are expressed as total cell  number × 10^4^.

### 2.8. Assessment of Th2 Cytokines in BAL Fluid Using Enzyme-Linked Immunosorbent Assay (ELISA)

The concentration of Th2 cytokines IL-4, IL-5, and IL-13 was measured using a quantitative sandwich enzyme-linked immunoassay kit (BD, San Diego, CA, USA, for IL-4,5 and R&D, Minneapolis, MN USA, for IL-13). A 96-well microtiter plate (Costar, NY, USA) was incubated overnight at 4°C with anti-mouse IL-4, IL-5, and IL-13 monoclonal antibodies in coating buffer, washed with PBS containing 0.05% tween 20 (Sigma, MO, USA) and blocked with 5% FBS in PBS and 1% BSA in PBS for 1 hour at 4°C and room temperature. 100 *μ*L of BAL fluids were then incubated for two hours at room temperature. Secondary peroxidase labeled biotinylated anti-mouse IL-4, IL-5, and IL-13 monoclonal antibodies were then incubated for 1 hour. Finally, the plates were treated with the TMB substrate solution (KPL, San Diego, CA, USA) for 30 min, and the reaction was stopped with the addition of the TMB stop solution (50 *μ*L per well). Optical density was measured at 450 nm in a microplate reader (SOFT max PRO, version 3.1 software, CA, USA). The detection limits for IL-4, IL-5, and IL-13 were 500, 1000 pg/mL, and 100 ng/mL, respectively. 

### 2.9. Determination of IgE Titers Using ELISA

For serum analysis, 96-well immunomicroplates (Costar, NY, USA) were coated with anti-mouse IgE monoclonal antibodies. The serum was diluted 1 : 250 with 5% FBS in PBS (assay diluent), and IgE levels (BD Pharmingen) were measured using standardized sandwich ELISAs according to the manufacturer's protocol. Optical density was measured at 450 nm in a microplate reader (SOFT max PRO, version 3.1 software, CA, USA). The detection limit of the IgE ELISA was 100 ng/mL.

### 2.10. Histological Examination

Trachea and lung tissues were removed from mice and fixed in 4% paraformaldehyde and then embedded in paraffin, after dehydration, cut into 4 *μ*m sections, and stained with hematoxylin and eosin (H&E) and periodic acid schiff reagent (PAS).

### 2.11. Statistical Analysis

Statistical analysis of the data was conducted using the Prism 5 software (GraphPad Software Inc., CA, USA). All values are presented as the means ± S.E.M. Differences between the means of the control and treatment samples were determined by one-way ANOVA or Student's *t*-test. Results with a *P* < 0.05 were considered statistically significant.

## 3. Results

### 3.1. EA Promotes an Increase in CD4^+^CD25^+^Foxp3^+^ Treg Cells

**Figure 3:**
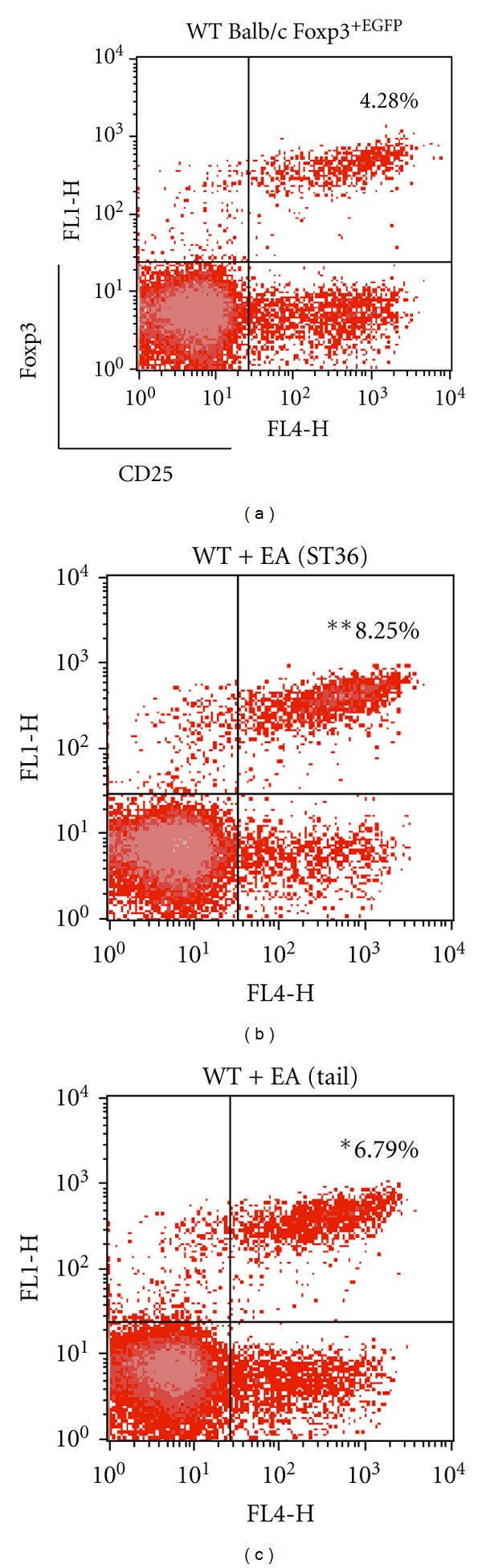
Flow cytometry analysis of splenic CD4^+^CD25^+^Foxp3^+^ Treg cells. WT Foxp3^EGFP^ mice were sacrificed after 10 days of EA stimulation. Isolated splenocytes were stained with anti-CD4 allophycocyanin (APC) and anti-CD25 PE and submitted to flow cytometry analysis. At first, CD4^+^ cells were gated (not shown), and then CD25^+^ and Foxp3 ^EGFP-positive ^ cells were analyzed. The average flow cytometry analysis data of CD4^+^CD25^+^Foxp3^+^ Treg cells (WT 4.28%; EA-treated at ST36 8.25%; EA-treated at tail 6.79%) were represented. Data are shown as mean ± S.E.M. (*P < 0.05; **P < 0.01 versus WT; *n* = 5).

To determine whether EA affects CD4^+^CD25^+^Foxp3^+^ Treg cells, EA stimulation at ST36 and a nonacupoint (tail), were applied for every 10 days to WT Balb/c Foxp3^EGFP^ mice (WT). Mice were sacrificed and splenocytes were then immunofluorescently stained with anti-CD4 allophycocyanin (APC) and anti-CD25 PE. CD25^+^ and Foxp3^EGFP-positive^ cells were analyzed using CD4^+^-gated cells. The EA at ST36 group increased the percentage of CD4^+^CD25^+^Foxp3^+^ Treg cells from 4.28% to 8.25% (*P* < 0.01) when compared with the WT group (Figure 3). The Treg cells in nonacupoint mice also increased from 4.28% to 6.79% (*P* < 0.01) relative to WT mice; however, the effect of EA at ST 36 was more significant. Therefore, EA induced a prominent increase of CD4^+^CD25^+^Foxp3^+^ Treg cells in the splenocyte cell population of WT mice.

### 3.2. The Effects of EA on CD4^+^CD25^+^Foxp3^+^ Treg Cells in OVA-Induced Asthmatic Mice and Treg Cell-Depleted Mice

**Figure 4:**
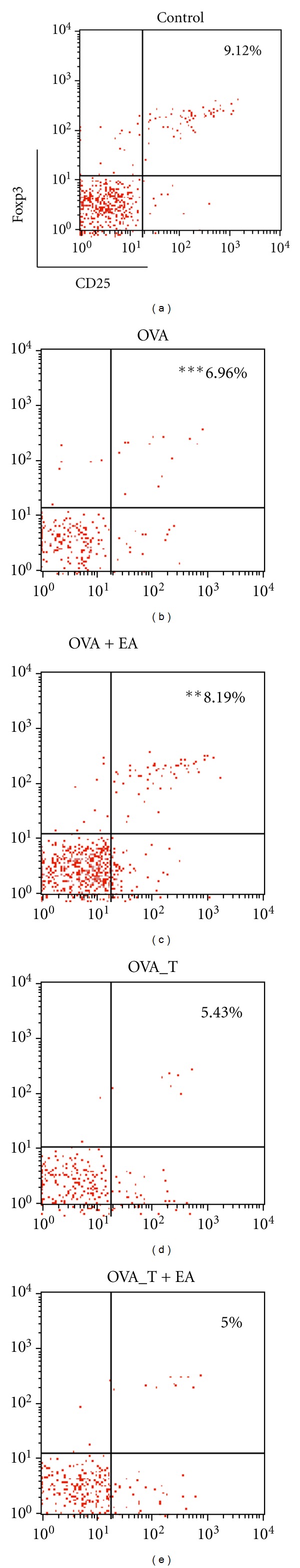
Effect of EA on CD4^+^CD25^+^Foxp3^EGFP^ Treg cells and Treg cells depletion in OVA-induced asthmatic pneumonocytes. PBS treatment alone (control); OVA induction only (OVA); EA treatment after OVA induction (OVA + EA); anti-CD25 Ab injection after OVA induction (OVA_T); EA stimulation after OVA induction and anti-CD25 Ab injection (OVA_T + EA). Isolated pneumonocytes were stained with anti-CD4 allophycocyanin and anti-CD25 PE and submitted to flow cytometry analysis. CD4^+^ cells were gated on and then CD25^+^ Foxp3^EGFP-positive^ cells were examined. The average flow cytometry analysis data of all groups were depicted. There was an increase of CD4^+^CD25^+^Foxp3^+^ Treg cells in OVA+EA group, but negligible data were shown in anti-CD25 Ab injected groups when compared with OVA group. Data are shown as mean ± S.E.M. (***P < 0.001 versus control; **P < 0.01 versus OVA; *n* = 6).

To evaluate the effects of EA on CD4^+^CD25^+^ Treg cells *in vivo*, the expression of CD4^+^CD25^+^Foxp3^+^ in Treg cells was analyzed in pneumonocytes of asthmatic Foxp3^EGFP^ mice. Flow cytometry analysis was conducted using pneumonocytes from OVA-induced asthmatic Foxp3^EGFP^ mice (OVA) that had been sacrificed 32 days after EA treatment. The pneumonocytes were then immunofluorescently stained with anti-CD4 allophycocyanin (APC) and anti-CD25 PE, and the Foxp3^EGFP^ population was analyzed. Among the asthmatic groups, the EA-treated group had a higher percentage of CD4^+^CD25^+^Foxp3^+^ Treg cells, which increased from 6.96% to 8.19% (*P* < 0.01) relative to the OVA group (Figure 4). We also examined the effects of injecting anti-CD25 Ab (PC61) on OVA-induced asthmatic mice to better understand the role of Treg cells in allergic asthma and to determine a possible relationship between Treg cells and EA. As shown in Figure 4, injection of anti-CD25 Ab did not increase the number of CD4^+^CD25^+^Foxp3^+^ Treg cells, although EA was conducted. These results suggest that EA treatment induced a significant increase of CD4^+^CD25^+^Foxp3^+^ Treg cells.

### 3.3. The Effect of EA on IgE Production and Th2 Inflammatory Cytokines Secretion

**Figure 5:**
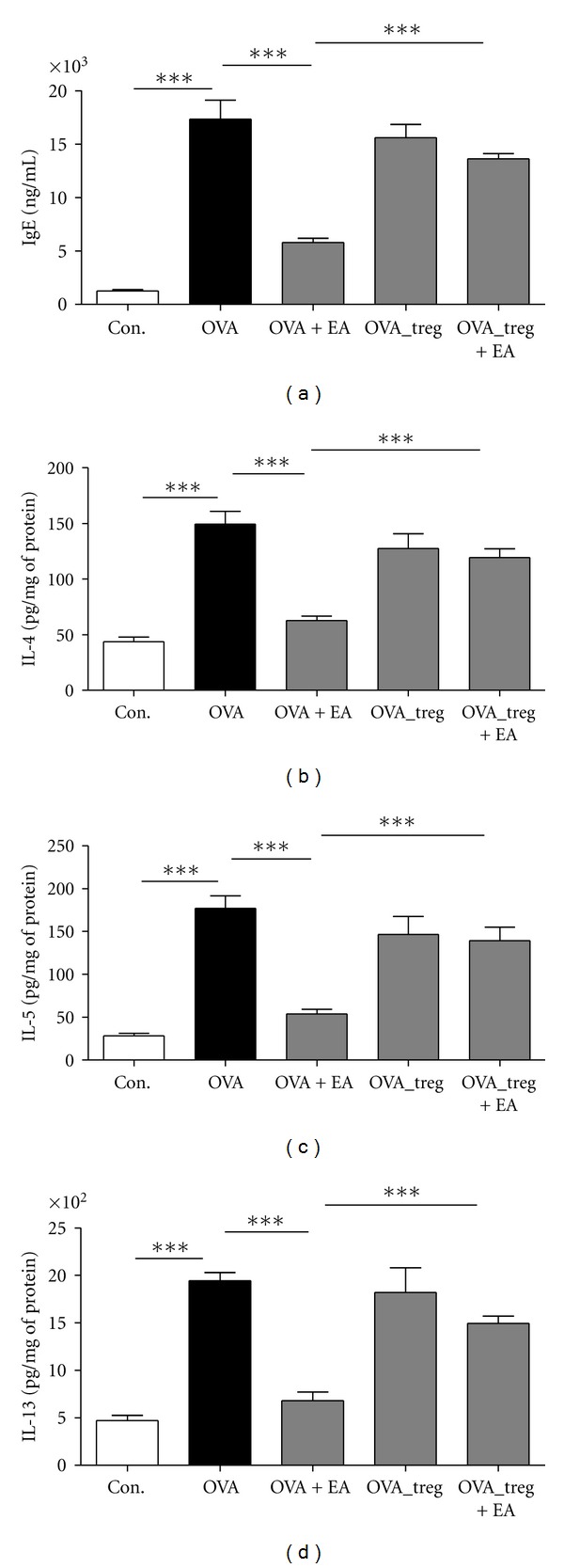
Total IgE production from serum and Th2 cytokines secretion from BAL fluid in OVA-induced asthmatic mice. Total IgE and Th2 cytokines (IL-4, IL-5, and IL-13) levels were measured by ELISA. The data shown represent the means ± S.E.M. Statistics were conducted using a Newman-Keuls multiple comparison test after one-way ANOVA. ***P < 0.001.

We also evaluated serum IgE production and Th2 inflammatory molecules generated by OVA-induced allergic asthma to ensure that EA decreased the levels of IgE and BAL fluid inflammatory cytokines, IL-4, IL-5, and IL-13 (Figure 5). Serum IgE and Th2 cytokines in BAL fluid were measured 48 h after the last EA treatment. Total IgE titers and secretion of cytokines, IL-4, IL-5, and IL-13 were significantly elevated in all OVA-sensitized and challenged mice when compared with the control mice. As expected, EA-treated mice had decreased levels of IgE (OVA 17350 ± 1775 ng/mL; OVA + EA 5795 ± 403.0 ng/mL), IL-4 (OVA 149.3 ± 11.57 pg/mg; OVA + EA 62.67 ± 4.092 pg/mg), IL-5 (OVA 176.9 ± 14.81 pg/mg; OVA + EA 53.81 ± 5.487 pg/mg), and IL-13 (OVA 1943 ± 86.81 pg/mg; OVA+EA 680.8 ± 91.12 pg/mg). However, Treg-depleted mice (OVA_T and OVA_T + EA) that received 0.25 mg of anti-CD25 Ab after OVA induction showed no significant differences in comparison to the OVA group, even though EA treatments were used.

### 3.4. The Effect of EA on Eosinophil Infiltration

**Figure 6:**
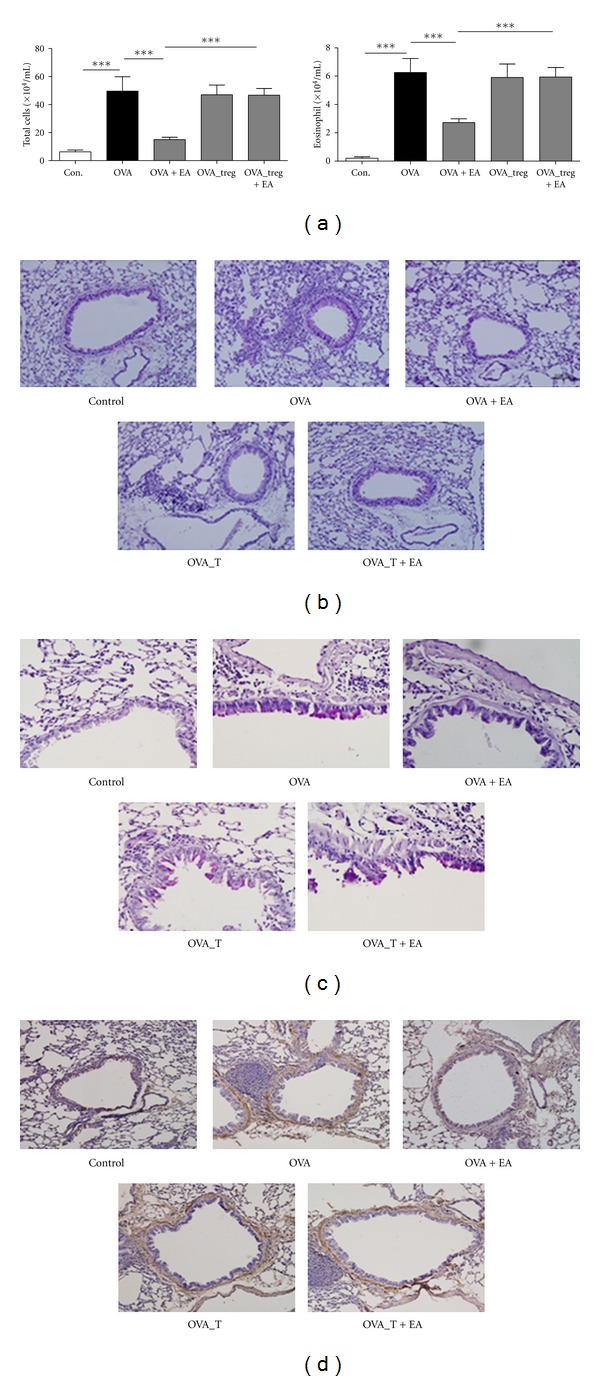
Effect of EA on various cell types in BAL fluid and lung tissue. (a) Total number of infiltrating cell and eosinophils in BAL fluid. (b) Inflammatory cell infiltrates in experimental group. (c) Goblet hyperplasia in experimental group. (d) Comparison of the thickness of thebronchiolar smooth muscle layer of experimental group. The data shown represent the means ± S.E.M. Statistics were conducted using a Newman-Keuls multiple comparison test after one-way ANOVA. ***P < 0.001.

 In the OVA-immunized animals, the total number of infiltrating cells was approximately 8 fold higher in the BAL fluid. However, EA treatment led to a 70% decrease in the total number of infiltrating cells when compared with the OVA group (Figure 6(a)). Eosinophils were counted based on their morphological characteristics and were visualized by Diff-Quick staining. The number of eosinophils in EA-treated animals was found to be a half of that in the OVA group (Figure 6(a)). Histological analysis of lung tissue sections revealed that the number of inflammatory cells, especially eosinophils, was significantly increased around the perivascular and peribronchiolar regions in the OVA group, the Treg depletion group and EA-treated Treg depletion group, but was decreased in the EA-treated OVA-immunized group (Figure 6(b)). The infiltration of inflammatory cells was virtually absent in the control group. And by extension, goblet cell hyperplasia and alteration in smooth muscle thickness was present in the OVA group and no significant difference was observed between the Treg depletion mice and EA-treated Treg depletion mice. In contrast, in the EA-treated OVA group, a prominent decrease in goblet cell hyperplasia and smooth muscle thickness was evident (Figures 6(c) and 6(d)). These data suggest that EA attenuated eosinophils and inflammatory cells in OVA-immunized mice through the modulation of CD4^+^CD25^+^Foxp3^+^ Treg cells.

## 4. Discussion

Asthma is one of the most common chronic disorders of the airways and affects adults and children of all age [23–25]. It has been estimated that worldwide, approximately 300 million people currently suffer from asthma, and about 180,000 deaths are associated with asthma each year [23, 26]. Allergic asthma is a complex disease that is characterized by reversible airway obstruction, elevated serum levels of IgE, chronic eosinophilic airway inflammation, airway remodelling, mucus hypersecretion, and AHR to bronchospasmogenic stimuli. Many asthma deaths are preventable, if treated with the appropriate medical care. Current standard medications involve combination therapies, including inhaled corticosteroids, *β*2-agonists, leukotriene receptor antagonists, and others [26, 27]. However, these therapies produce potential side effects, such as retardation of growth, loss of bone mass, induction of insulin resistance, and suppression of the immune system, and do not consistently ameliorate airway inflammation in many asthmatic individuals [24, 28, 29]. Therefore, there is a need for the development of safe and efficacious treatments [30, 31].

Clinical and experimental studies have shown that sequential EA stimulation was effective in the treatment of stress-induced immunodeficiency and physical disorders [32]. In recent meta-analysis study demonstrated that pharmacoacupuncture, a combined therapy with acupuncture and herbal medicine, possessed potential beneficial effects on asthma [33]. Even though there were some debates about placebo effects of EA, recent systemic review suggested that the acupoint stimulation elicited more specific responses than sham acupuncture [34], in which similar effects were also shown in the present study.

Electrical stimulation can effect physiological systems, which include induction of the secretion of cytokines, activation of NK cell activity, and the release of opioid peptides both in the periphery and the central nervous system [35–37]. This investigation was based on our previous work, where we found that EA regulates the expression of IgE and Th1/Th2 cytokines involved in DNP-KLH immunized mice [11, 12], and some of the studies and clinical experiences have shown that EA reduces the inflammatory response during allergy pathogenesis in an OVA-sensitized model and rheumatoid arthritis [2, 3, 38]. Therefore, we investigated the effects of EA on CD4^+^CD25^+^Foxp3^+^ Treg cells and the relationship between acupuncture mediated antiallergic effects and Treg-cell responses in a murine model of asthma. To the best of our knowledge, this is the first study where acupuncture-mediated anti-allergic asthma was evaluated in regards to the modulation of CD4^+^CD25^+^Foxp3^+^ Treg cells.

Th1 cells are involved in delayed-type hypersensitivity and cytotoxic response, while Th2 cells regulate allergic diseases by activating B cells and regulating IgG and IgE secretion [39]. Specific IgE production is a hallmark of allergic diseases [40, 41]. In particular, IL-4 or IL-13 is essential for the first step in IgE production. In addition, IL-13 is both necessary and sufficient to induce AHR. Other cytokines including IL-5 and IL-6 can enhance IgE production [42–46]. In the present study, ST36 EA stimulation suppressed the increased antigen-specific IgE production and Th2 cytokines such as IL-4, IL-5, and IL-13 in OVA-induced allergic asthma mice. IL-5 is specifically involved in the development, priming, and survival of eosinophils [47]. Furthermore, eosinophils, basophils, and some structural cells such as epithelial and endothelial cells may also produce cytokines and chemokines that amplify the inflammatory cascade [48, 49]. In addition, infiltration of eosinophils in the airway results in the abnormal production of inflammatory proteins and cytokines, such as eosinophil cationic protein, major basic proteins, leukotrienes, IL-4, IL-5, IL-6, IL-13, and TGF-*β* [24, 25, 50]. It has previously been shown that in the OVA-immunized asthma model, AHR is closely associated with eosinophilia, as evidenced by an increase in the number of eosinophils in BAL fluid [13, 18]. The results of the present study indicated that EA stimulation led to increased CD4^+^CD25^+^Foxp3^+^ Treg cells, whereas peribronchial and perivascular inflammatory cell infiltrates were significantly suppressed.

In summary, we investigated the effects of electroacupuncture on CD4^+^CD25^+^Foxp3^+^ Treg cells and the relationship between acupuncture mediated anti-allergic effects and regulatory T-cell responses in OVA-induced asthmatic mice. EA stimulation attenuated the pathogenesis of allergic asthma in an experimental mice model. The production of serum IgE and Th2 cytokines in OVA- induced EA-treated group were less than those in the OVA group. Also, the total number of inflammatory cells and the infiltration of eosinophils was reduced in EA-treated mice. The result presented here indicate that sequential ST36 EA stimulation promotes CD4^+^CD25^+^Foxp3^+^ Treg cells and suppresses the increase in IgE production and Th2 cytokine secretion in OVA-induced asthmatic mice presumably through the modulation of Th1/Th2/Treg cell balance. Our results also imply that EA can modulate Treg cells to downregulate Th2 cytokines and infiltration of inflammatory cells, and EA stimulation has strong immunomodulatory effects on CD4^+^CD25^+^Foxp3^+^ Treg cells, moving toward an immunologically balanced state to maintain homeostasis.

## 5. Conclusions

Taken together, the identification of EA stimulation-induced CD4^+^CD25^+^Foxp3^+^ Treg cells will help elucidate the mechanism of acupuncture-mediated anti-allergic effects, which will open a new route for therapy and may provide new methods of treating allergic disorders.
